# Complimentary Banquet to Dr. John P. Buckley

**Published:** 1922-02

**Authors:** 


### COMPLIMENTARY BANQUET TO DR. JOHN P. BUCKLEY

The Southern California Dental Association, October 8, 1921, extended a banquet to Dr. J. P. Buckley, president-elect of the National Dental Association, in Los Angeles.

It was largely attended by prominent dentists of California, who contributed generously and fittingly to the occasion. We are so impressed with the replies made to the toasts by Dr. Buckley that we reprint in part his remarks. Those who know him personally will appreciate what he said and why he made such a naive and sympathetic address to his newly-formed friends. His old Chicago neighbors expected an earnest and affectionate tribute to things always dear to him, and they are delighted to know that he has so captivated his new neighbors. We trust and hope his new environment and improved health may amply fortify him for the strenuous task that is now before him.

While I fully realize my unworthiness of the many kind things which have been said, and while each speaker has been more than generous in his praise of my feeble efforts in the past—it seems that each of them purposely couched his language in terms which has had the effect of cheerfulness for me rather than of depression, which might easily have been the case. For this I am truly grateful, and because of this I am tempted to speak at greater length than I would have done, had conditions been different

As I sat here tonight and listened to the speakers, my mind turned backward in an effort to remember when and how I first met each of them. It is said that when one begins to reminisce it is an indication of age. Be that as it may, I shall hold you responsible for anything I may say under the spell of the moment. I do not remember just when I first met Dr. John Millikin. I first heard of him through his services as a dental surgeon in the U. S. Army. I remember he stopped in Chicago four years ago on his way to the New York meeting of the National. He was present at a dinner of a small group of men and later spoke before the Chicago Dental Society. It was at New York where he was first elected to the trusteeship of the National. I don't know why you men here in the west selected him for this important position. I was not so much interested in things here then; but as a member of the board of trustees for years from the sixth district, comprising Illinois and Wisconsin, I do know that this ninth district had not received much recognition in that body. This was the fault of no one but yourselves; for your previous trustees were seldom at the National meetings. Since his election four years ago, Dr. Millikin has not missed a single meeting of the National Association. He has been present at the meetings of the board of trustees; and so I am not at all surprised when it came time to elect a trustee from this district at Milwaukee recently, that Dr. Millikin received the honor without opposition. He was the only one of the three trustees whose term expired this year who succeeded himself. It takes time for any man in the board of trustees to get acquainted with the workings of the National, and while Dr. Millikin served you well during his first term, he is in a better position now to do more for dentistry here in the west than he could possibly have done heretofore. Dr. Millikin never overlooks an opportunity of doing all he could for those whom he had the honor to represent. In other words, he is a real, live, energetic representative, and well you can be proud of him.

The first time, I think, I ever heard of Dr. Jas. D. McCoy was one evening about six or seven years ago, when I went to the dental college in Chicago to deliver my weekly lecture. We had recently installed a new X-ray machine, and I was arranging to have the students check up on their root-canal work by means of the X-ray. The secretary of the college had charge of the new machine and took the pictures for the students; and this evening as I walked in he was reading a book in which he seemed deeply interested. When I asked what he was reading he showed me the book and said it was one of the best works he had seen for students and practitioners on dental radiography. I glanced at the book and learned that it was written by Dr. Jas. D. McCoy of Los Angeles. Since I have been here I have found that this man has done more for dentistry than write this book—valuable as that has been. You haven't a man among you whose ideals are higher or who has a greater regard for the ethics of his profession than Dr. McCoy, and I deeply appreciate the kind things he has seen fit to say on this occasion.

Nearly 20 years ago I was on my way home from New York City, where I had read a paper, and stopped for a day at Philadelphia. Among other dentists I called on Dr. E. C. Kirk, the editor of the *Dental Cosmos.* I always considered the *Dental Cosmos* one of the best journals published. Dr. Kirk's editorials were classics; and for some time past I had taken an unusual interest in the department of the journal called the ''Periscope'' and the ''Review of Current Literature,'' which was conducted by a man whom I had never met. His name was Julio Endelman. I did not know at the time whether he was young or old, short or tall, slim or stout. All I knew of him was that he had a characteristic style of writing—one all his own, and from his writings he seemed to be interested in things pathologic and in the therapeutics of dentistry. This naturally attracted me, and here in Dr. Kirk's office, as I say, nearly twenty years ago, I first met Dr. Endelman. After a few years I missed him from the pages of the *Dental Cosmos,* and when I came here in 1915 on my way to the Hawaiian Islands, previous to the convening of the Dental Congress at San Francisco, I found to my surprise that Dr. Endelman was here. Not long ago he told me that if some one in authority should decree that he could never leave California again, he would be perfectly satisfied. He, too, came west in search of health, and he found it. As a reader, a thinker, and a writer, as a student and a teacher, Dr. Endelman has few equals in his profession; and it is easy for me to see why you appreciate him here as you do.

I was always interested in things chemical, and for some years I taught chemistry and metallurgy in the Chicago College of Dental Surgery. In our metallurgical experiments we followed the text as found in the book on

Dental Metallurgy, written by Dr. Jos. D. Hodgens, of San Francisco, and for years Dr. Hodgens and I have been friends largely because of our mutual interest in chemistry. In later years I noticed that this book was revised by a Dr. Guy S. Millberry. From the first edition Dr. Hodgens' book on Dental Metallurgy was the best published; and while it seemed difficult for any one to improve on the work, Dr. Millberry's efforts at revision, working as he did conjointly with the author, accomplished this difficult task. Did you notice how his thoughts tonight ran along lines chemical and metallurgical? I do not recall when I first met this forceful character. But I do remember distinctly the first time I ever heard him speak in public. It was at a banquet given in the Oakland Hotel following an intensive course which I had given in root-canal work before the Alameda County Dental Society in February, 1917. At this banquet Dr. Millberry asked me if I would come to his school and talk to his students. Always enjoying my work with students, I gladly accepted. On the way to the college I asked him what I should talk about and he said he would like to have me consider the treatment of pulpless teeth—it was in the early days of focal infection—and the indications and uses of formocresol. I told him that I had better not do this, for I was not familiar with what had been taught by his professor of therapeutics and might say something which would conflict. I remember well Dr. Millberry's reply. He turned to me and said with that earnestness so characteristic of the man: "Dr. Buckley, when we get to the point in our college where we are not willing to have our students hear both sides of a proposition of interest to them so that they may judge for themselves, I want to resign the deanship of the college." I met a large body of students—the three classes must have been assembled—and talked for over an hour on my pet subject, and never did I enjoy meeting students more. They listened with that seeming interest which commanded respect.

Now what shall I say about the toastmaster, Dr. Bert Boyd. I do not remember when I first met him; but since I have been here I have learned to know him well. In many ways he reminds me of one of my many Chicago friends, Dr. Thos. L. Grisamore. Dr. Grisamore was my classmate in pharmacy and both my classmate and roommate in dentistry. We have ever been the best of friends. I must find in you men here a corresponding friend of the men in Chicago or I just could not stay. This fortunately I have been able to do; and Dr. Boyd reminds me in many respects of Dr. Grisamore. If he knew Dr. Grisamore as I know him, he would realize I could pay him no greater compliment.

This, my friends, brings me to say something as to why I am here and whether I intend to stay. If I were perfectly well tomorrow I would remain in California. A great many of my friends back east do not understand this. I can scarcely understand it myself. There are some things in this world which do not worry me if I fail to understand. This is one of them. I do not want to be misunderstood here. This does not mean that I have forgotten the city of Chicago or the state of Illinois. It does not mean that I have willingly or otherwise permitted you men to take the place of the good friends I have in Chicago or in Illinois. No one will ever replace in my life and heart those great and good men with whom I was privileged to associate for over twenty years. Let this be plainly understood. I could be the most unhappy man today in the dental profession, but I am not. I was forced by reason of ill-health to leave an independent practice among the best people in Chicago and surrounding country; a college position which I had held for years and which afforded me the opportunity to experiment along lines in which I was deeply interested; yes, to leave professional and social friends, the like of which no man ever possessed; to leave the place where our only child was born and which had long been our home; and to come to a strange land among more or less strange people. Who are those men whom I left in Chicago? Brophy, Johnson, Case, Gilmer, Taggart, Gallie, Logan, Goslee, Noyes, Black, Dittmar, Roach, Elliot, Grisamore, Prothero, Moorehead, Coolidge,—oh! I could go on and on and on. To name one is but to name them all. These men are the ones about whom you read; in many instances they are the authors of our text-books; and they were my daily associates. I was free to consult them on any and every occasion. It was with them that I lived, and fought, and loved; and just when I had reached the goal where any of you would liked to have been, I was forced to leave; so I think you can understand how easy it would be for me to be the most unhappy man in the profession today—a strange man here among what might be strange men. Yes, what "might be," but what *is* not. You men have taken me in with all of my peculiarities and faults, and I have many of them; you have made me one of you, and in so doing you have made me happy. I have lived long enough to know that not all of the so-called pleasures of life bring happiness; that there is a vast difference between the pleasures of life and true happiness. Happiness comes only from the consciousness of realizing ideals.

This week, as you know, the American Bankers' Association has been in session here. Tuesday evening we had at our men's Club of the First Presbyterian Church of Hollywood a prominent banker from New York City. He was supposed to speak according to the program on "Finance"; but it developed that he talked on everything except money matters, and no one present was disappointed. This man began his banking career in Muskogee, Okla. During his discourse he spoke of traveling on one occasion with a multi-millionaire of New York City, and on the trip the latter asked him what he thought a man must have to be rich. Our speaker replied that, in his opinion, it would depend largely on where the person lived; that if he lived in Muskogee and had a hundred thousand dollars, he would be considered rich; in Dallas, Texas, for instance, he probably would need to have two or three hundred thousand; and in Los Angeles, perhaps a million dollars; but in Chicago or New York City, it would require several million before a man would be considered rich. Then he said, I have given you my answer to your question, may I ask you what you think a man should have to be rich? The reply of the multi-millionaire was as brief as it was surprising. He said, "Happiness and contentment." Here in California I am rich for I am happy and contented. Now I want you to know that I fully appreciate there are different kinds of contentment. There is a contentment which leads to stagnation. This is the dangerous kind and should be avoided. The kind of contentment I mean is that which enables you to fit in with your environment and permits you to grow with those with whom you associate. This kind of contentment, and this kind only, is truly worth while. It was Emerson who said:

> "We came into this world like brother and brother,
> Now let us go hand in hand, not one before the other."

With these thoughts in mind, I can say to you again that here in California I am happy and contented.

Dr. Millberry referred to the dedication of my book—it being dedicated to my mother and to my wife—to my mother for the encouragment in my boyhood days; to my wife for the many sacrifices so willingly made in the earlier years of our married life. In thus dedicating my book I did only what each of you would have done under similar circumstances. Each of you must remember those "gentle droppings from a mother's lips which day by day and hour by hour grow into the enlarging stature of one's soul and live with him forever"; and the sacrifices our wives make that we may meet with success in our life's work! With me these sacrifices were always made with that grace and willingness which gave encouragement; and if I merit in even the smallest degree the things which have been spoken here tonight, I owe it largely to my little helpmate. In your thoughtfulness regarding this banquet you did not overlook an invitation to my wife and son, and I thank you for this. This banquet should be given to them—not to me.

Would I dare relate a personal incident regarding my son? I remember one day—it was a beautiful summer day when all nature beckoned one to come out and enjoy God's sunshine, I had planned not to go to the office in order that I might complete a chapter for the book which I had well in mind. Clarence had come in the library several times during the day, being all alone and no one to play with, and each time I would put him out—perhaps I was not always as pleasant about it as I should have been. I was anxious to get the book finished and had certain things definitely in mind. With me, I can not write unless I can think, and I can not think unless I can concentrate, and I can not concentrate unless I am alone; therefore on this occasion I wanted to be alone, and each time Clarence would come in I would thoughtlessly put him out. Late in the afternoon he came in again, he was not crying nor did he complain—he only had a sad expression on his little face which I shall never forget, and looking up at me he feelingly said: "Papa, I wish you did not have to write so much so that you could play with me." When the book was finally finished and I was ready for the dedication, I felt like referring to this incident also; but of course it was too personal for publication. Nevertheless, on the flyleaf of the first book which came off the press, I wrote:

"To Clarence E. Buckley, who so often came into my library and said, 'Papa, I wish you did not have to write so much so you could play with me'—this book—the first copy—is presented by the author."—Signed, J. P. Buckley, November 20, 1909.

For me to have been able to spend more time with my wife and son here in California than I was seemingly, at least, able to do in Chicago, has not detracted from the happiness and contentment which I have found here.

Now, my dear friends, I have talked far too long, and I have mentioned things far too personal for public utterance; but your attention and earnestness tempted me and I yielded to the temptation. I want to thank all who in any way are responsible for this pleasant evening—one long to be remembered by myself and family; and I only hope that I may live long enough to convince you that while wholly unworthy, I nevertheless appreciate your good intentions. Strange as it may seem to you, I have really enjoyed hearing these kind words. I thank you from the bottom recesses of my heart.—Extract taken from *The Pacific Dental Gazette.*

